# Case of Fracture of the Skull,—Compression of the Brain—Laceration of the Dura Mater—Escape of Cerebral Substance—Depression of the Bone—treatment—recovery—and Remarks

**Published:** 1843-06-24

**Authors:** John S. Rohrer


### THE MEDICAL EXAMINER,

### And Retrospect of the Medical Sciences.

Vol. VI.] PHILADELPHIA, SATURDAY, JUNE 24, 1843. [No. 12.

### CASE OF FRACTURE OF THE SCULL,

*Compression of the Brain—Laceration of the Dura Mater—Escape of Cerebral substance—Depression of the Bone—Treatment—Recovery—and Remarks.*

By John S. Rohrer, M. D.

October 16, 1842, John Francis Gorman, aged eight years and six months, was thrown from horse back, while the animal was in full speed, and dashed upon the pavement. His forehead struck a stone, and, at the same instant, the horse trod upon the right side of his head. He was picked up in a state of insensibility, and conveyed to his father's residence, in Schuylkill Sixth street above Chestnut, a short distance from the spot where the accident occurred. I was called to see him at 3 o,clock, P. M., a few miuutes after he received the injury, and found him labouring under symptoms of compression. His skin was cold, the limbs relaxed, and pulse almost imperceptible. The pupils of his eyes motionless and *slightly contracted.* His breathing was slow and interrupted, and the motion of the heart feeble. After having had him removed from the crowd which pressed upon him, and conveyed to his bed, there were slight contractions of the muscles of his arms, and a portion of froth escaped from his mouth.

Upon examining his head I discovered an extensive fracture of the lateral portion of the parietal bone extending backwards across the lamboidal suture of the occiput. The integuments were cut and contused, with a large portion of bone laid bare, and a considerable flow of blood. The external wound was *two inches in length,* whilst the *fracture* was about *three inches across*, and the bone driven on the brain *one inch below the surface.* The *dura mater* was cut through, and upon removing the coagulated blood and loose hair from the wound, about *two teaspoonfuls of cerebral matter*, mixed with blood, escaped from the opening of the scull.

The bone having sunk about an inch below its level, I introduced a lever and elevated it as far as practicable, *without doing violence* to the parts. By this procedure the pressure of the brain was removed, and vomiting immediately followed, after which sensibility and consciousness returned. The fractured portion of the bone was still much depressed, causing a large indentation in the form of a semicircle, the opening into the brain being on the straight line forming the diameter of the half circle.

There was also found a transverse fissure of the *os frontis*, about two inches in length, with a wound of the integuments, and considerable ecchymosis and tumidity of the parts, closing entirely one eyelid.

After the partial elevation of the depressed portion of bone, the patient, as I stated before, immediately recovered his sensibility, and was much relieved, but soon afterward relapsed into a state of *drowsiness* and *stupidity.* Whether these symptoms were the effects of concussion or commotion which the brain had sustained from the injury, or whether compression was about taking place in consequence of accumulation of fluid beneath the fracture, could, at this time be but a matter of conjecture. The fact, however, that he was sensible to the slightest irritation of the wound, and could be aroused from his state of lethargy when spoken to in a loud tone of voice, were strong reasons for believing that the application of trephine, at this period, would not only be premature, but might be attended with very bad consequences. The case being as thus presented, and especially considering the fact of the depressed bone being nearly an inch below its surface, not only were the utmost care and deliberation necessary on the part of the surgeon, but there devolved upon him an extremely high degree of responsibility, as to the course of treatment to be adopted and pursued.

My friend, Doctor Mütter, who was called in consultation at this juncture of the case, at first urged the necessity of an operation, for the purpose of relieving the existing bad symptoms, as well as to remove the depressed portion of bone, but upon being informed of the temporary relief obtained, from the partial elevation of the bone by the lever, and finding the patient sensible to pain, and more conscious of his situation, when perfectly aroused, than he had anticipated, the Doctor coincided with me in opinion, and agreed to postpone the operation, until more urgent symptoms should point out its necessity. In having recourse to the *trephine* in the present instance, two objects only, I conceived, could be aimed at; *first*, to elevate the depressed portion of bone, and, *secondly*, to give room for the escape of blood and matter which might accumulate, to prevent subsequent pressure on the brain. In respect to the latter, no possible good could result from an operation, as the opening of the scull was large enough for the passage of a quantity of *blood and cerebral matter*, which had already escaped. I am aware that the operation of *trephining* has frequently been performed for the purpose of removing depressed portions of bone, when it was not contended that symptoms of compression were present. But such practice, in my opinion, is injurious, and can never be justified, where there is an *opening in the scull for the transmission of blood and matter*, unless in cases where spiculæ of bone are driven on the brain, and even then the utility of an operation is doubtful, if they cause no irritation, and their precise situation is not easily defined. Too much precipitancy, therefore, on the part of the surgeon with the use of the *trephine* is frequently attended with great harm. It is not the use, but the abuse of the trephine that I deprecate. No surgeon can be too careful in making up his mind as to the course he pursues in the treatment of fractures of the cranium. Hasty decisions, without due investigation into the real nature of the case in question, cannot be too severely reprehended. Patients are often subjected to cruel and unnecessary operation from fractures of the scull; and in many cases such operations have proved more dangerous than the injury itself. Numerous instances of unwarrantable interference of this kind could be cited, but as their publication would neither benefit or edify the profession, I shall decline giving any.

Having long entertained the above views in reference to cases of this sort, and being fully convinced that the *trephine* could not be used with good effect in the present case, I concluded, inasmuch as Dr. Mütter coincided with me, not to apply it, unless more urgent symptoms than the above described should supervene, to warrant such interference. Considering the welfare of the patient to depend, in a great measure upon this step alone, and being aware of the importance that many surgeons attach to the immediate application of the trephine in similar cases, I received, with great satisfaction, the favourable concurrence of Dr. Mütter on this important point in the treatment of the patient. Having understood that high authority had animadverted on this subject, and denounced, in unequivocal terms, similar treatment, regarding the *depression of bone*, as always giving rise to epilepsy or other bad consequences, I will briefly add, that although such lesions unquestionably are to be deplored, their occurrence does not necessarily lead to the unfavourable consequences above alluded to, and that numerous instances might be adduced where extensive fracture and depression of bone gave rise to no subsequent ill health to the patient. I contend, moreover, that in cases of fracture, the elevation of depressed bone, if to be effected only by great force, even if practicable, would do more harm than good.

The *trephine*, to which a few dazzling examples of success are ascribed, and which have instilled in the young surgeon an immoderate solicitude to operate, would, in similar cases, be a useless instrument, unless, he proposed to bore round the depressed bone, like a carpenter would in removing a plug from a piece of timber. *So large a surface of bone* depressed as above described, could not be brought to its proper level, where the ordinary means of the lever failed, without entirely detaching it from the surrounding bone, which, in the present advanced stage of medical science, I trust no one would attempt. Without further entering into details I will briefly give the opinion and experience of some of the most eminent surgeons, who have written on this subject. M. Abernethy relates several cases of fracture of the cranium with compression, which terminated favourably, although no operation was performed. In *Mr. Hill's cases of surgery* two instances of this sort are related, and Mr. Hill knew both patients for many years afterward; yet no inconvenience arose. Dr. Cooper says, "indeed it is not easy to conceive that the pressure, which caused no ill effect, at a time when the contents of the cranium filled its cavity completely, should afterward prove injurious, *when they have adapted themselves to its altered size and shape.*" * The practice of trephining with a view of *preventing* bad consequences as recommended by Pott, has been strongly remonstrated against by Messrs. John Bell, Desault and others. The patient under consideration retained his senses after the partial elevation of the bone, and Mr. Abernethy gives it as his opinion that "whenever the patient retains his senses perfectly he should think it improper to trephine him, unless symptoms arose that indicated the necessity of it." † If the citations which I have given from these eminent authorities, in addition to the favourable issue of the case in consideration, will serve to caution the reader against the too hasty interference with the trephine in similar instances, my object in reporting the case will have been fully attained. The subsequent treatment was as follows:

After the patient was put to bed a large poultice of bread and milk was applied over the wound.

6 o'clock, P. M. Patient is sensible, but indifferent to surrounding objects; pulse moderately full; skin warm and comfortable; pupils dilated and sensible to light.

10 o'clock, P. M. Pulse full, with some degree of tension; restless, and complains of pain of the head; skin dry and hot; calls for water. Bled him twelve ounces from the arm, which produced a strong impression on the heart and arteries, sickness of the stomach, and moisture of the skin. Ordered half an ounce of salts, to be followed, if necessary, by an enema.

11 o'clock, P. M. Rests well; pulse soft; but more frequent; skin moist, and thirst abated.

October 17. 9 o'clock, A. M. Patient composed until after midnight, when fever supervened. Had been delerious at times during the night; complains of pain and giddiness of his head; pulse full and strong; starts in his sleep. Bled him six ounces from the arm.

9 o'clock, P. M. Rested well since morning; skin moist; tongue clear; no pain in the head or delirium; thirst abated; medicine operated on the bowels. Poultice continued; medullary matter discharged from the wound

18th. 9 o'clock, A. M. No excitement; pulse regular and soft; tongue moist and clean; considerable quantity of blood and matter discharged from the wound.

19th. 10 o'clock, A. M. A lively expression of the eyes; slept well during the night; craves for food. Small quantity of bread and boiled milk allowed him. Bowels open, healthy evacuations. A small quantity of *medullary matter* discharged from the wound. Poultice continued.

9 o'clock, P. M. Patient doing well, no unfavourable symptoms.

20th. 9 o'clock, A. M. Rested well during the night, wound discharges freely. Appetite good; pulse soft and regular; sits up in bed.

4 o'clock, P. M. Patient's general health very good.

21st. A. M. Copious discharge from the wound; dressing composed of lint, removed three times a day. Too much fulness of the pulse; a small quantity of the solution of salts administered.

9 o'clock, P. M. Medicine operated freely; excitement moderated.

22d. A. M. General health good, wound doing well.

23d. Patient's condition the same; dressings continued as before.

Nov. 1st. The patient's general health continuing good; wound healing slowly; considerable discharge. Dressings consist of lint and Basilicon ointment.

10th. Wound closing slowly; fungus appearing; dressings as before, with the addition of hydrarg. oxyde rub.

1843. Jan. 15th. Patient called at my office this day, removed a *spicula of bone* from the wound; fungus reduced.

20th. Wound closing fast. Health good, no giddines or unfavourable symptoms of any kind.

Feb. 27th. Patient called at my house; wound nearly closed; intellect *not the least impaired*, and health entirely restored.

---

* Doctor Samuel Cooper's Dictionary of Practical Surgery, volume 1st., page 466.

† Same book, page 467.

March 7th. A spicula of bone removed from the wound. Patient has been at school and enjoys good health.

19th. One more *spicula of bone* removed, larger than the foregoing, presenting in patches on each surface the tables of the bone.

25th. Upon calling upon the parents of the child, and interrogating them respecting their son, they informed me that "his health is perfectly good, his mind clear, his intellect as bright as they have ever known it; that he has been at school for several weeks, and that he performs as much mental labour as he ever did, without any detriment."

May 2d. More than six months have now elapsed since the occurrence of the injury; and it is with no ordinary feelings of pleasure, that I am enabled to announce the entire restoration to perfect health, both physical and *mental*, of my patient. This result is the more satisfactory, as recoveries in cases of similar magnitude are very rare, and especially, in connection with the loss of MEDULLARY SUBSTANCE.

